# Supplementary material for: Consensus-based guidance for conducting and reporting multi-analyst studies
Source: eLife. 2021 Nov 9;10:e72185. doi: 10.7554/eLife.72185 (PMC8626083; doi:10.7554/eLife.72185)
Supplement: Supplementary file 1. [file elife-72185-supp1.docx]

**Appendix 1** - Reporting Template for Multi-Analyst Studies

The following template was created to support the reporting of multi-analyst projects. The present document focuses on methods and details of the multi-analysis process itself.

As multi-analyst projects can differ along many dimensions, this template should be freely modified according to the parameters of the specific projects. Furthermore, this document could also serve as a checklist on what to include in the methods section of multi-analyst projects.

## Reporting Template

We aimed to recruit a minimum number of ___ in order to check the robustness of the conclusions / assess variability of analyses.

Co-analysts were recruited using the following sources: ___

The eligibility criteria for our co-analysts were the following: ___

In total, ___ co-analysts signed up to the project, ___ of whom met the eligibility criteria. ___ of the co-analysts came from *Source 1*, ___ from *Source 2 ...*

The lead team also *conducted / did not conduct* an independent analysis on the same research question as the co-analysts.

After the recruitment, the co-analysts were informed about the following:

(a) their tasks and responsibilities;

(b) the project code of conduct (e.g., confidentiality/ non-disclosure agreements);

(c) the plans for publishing the research report and presenting the data, analyses, and conclusion;

(d) the conditions for an analysis to be included or excluded from the study;

(e) whether their names will be publicly linked to the analyses;

(f) the co-analysts’ opportunities to update or revise their analyses;

(g) the project time schedule;

(h) the nature and criteria of compensation (e.g., authorship, monetary rewards, etc.);

(i) why and at what stage co-analysts are allowed to communicate about the analyses (e.g., to catch errors or call attention to outlying data points).

In order to ensure independence, all members of the ___ co-analyst teams *signed an agreement/agreed* that they would not publicize, release, or discuss their analysis choices and the results with anyone until after all initial reports have been submitted. The non-disclosure agreement is available at _____________.

After accepting the conditions of participation, co-analysts were provided with the research questions and the dataset(s) accompanied with a codebook that contained a comprehensive explanation of the variables and the datafile structure on 20__ -__ -__. The research questions were provided without communicating any preferred analysis choices and or expectations about the conclusions. The dataset along with the accompanying codebook is available at _____________. To ensure that the co-analysts understand the restrictions on the use of the data, including issues of ethics, privacy, confidentiality, or ownership, we _____________.

The deadline of the co-analysts to submit their analysis code with explanatory comments (or a detailed description of their point-and-click analyses), their conclusions, and an explanation of how their conclusions follow from their results has been 20__ -__ -__.

Out of the ___ participating teams, ___ submitted their work by this deadline. An additional ___ teams submitted their work by an extended deadline of ____ .

Out of these submitting teams, ___ teams consisted of ___ members, ___ teams consisted of ___ members…

Out of the ___ co-analysts, there were ___ senior researchers, ___ post-doctoral researchers, ___ graduate students, and ___ members from other positions.

Out of the ___ submitted analyses, ___ were withdrawn, and ___ were omitted from the summary analysis for the following reasons: …

From ___ co-analyst teams who submitted their work, we received ___ analyses and results. The lead team made the commented code, results, and conclusions of all non-withdrawn analyses publicly available *before / at the time of* submitting the research report.

The submitted code, results, and conclusions were processed the following way: ___

The summary of the results of the co-analysts are: ___

The limitations and potential biases of the study are as follows: ___

[A practical way to summarize the analytic approaches and results is to present them in a table or graph. Before approving the publication of the study, the co-analysts could add their reflections on the project or its results. These comments can supplement the report of the study.

Some previous examples for summarizing and visualizing the results are [here](https://www.sciencedirect.com/science/article/pii/S002239992030773X) and [here](https://link.springer.com/article/10.3758/s13423-017-1417-2/tables/4).]
