# Supplementary material for: Consensus-based guidance for conducting and reporting multi-analyst studies
Source: eLife. 2021 Nov 9;10:e72185. doi: 10.7554/eLife.72185 (PMC8626083; doi:10.7554/eLife.72185)
Supplement: Supplementary file 2. [file elife-72185-supp2.docx]

**Appendix 2** - Reporting Checklist for Multi-Analyst Studies

Reporting Checklist for Multi-Analyst Studies

| IItem No | Recommended reporting item | Page number(s) where item is reported |
| --- | --- | --- |
| 1 | Justification for the number of co-analysts |  |
| 2 | Eligibility criteria and recruitment of co-analysts |  |
| 3 | How co-analysts were given the data sets and research questions |  |
| 4 | How the independence of analyses was ensured |  |
| 5 | Numbers of and reasons for withdrawals and omissions of analyses |  |
| 6 | Whether the lead team conducted an independent analysis |  |
| 7 | How the results were processed |  |
| 8 | Summary of the results of co-analysts |  |
| 9 | Limitations and potential biases of the study |  |
| 1 | How the reader can access the data and code for all analyses |  |
